# Calcium and fat metabolic balance, and gastrointestinal tolerance in term infants fed milk-based formulas with and without palm olein and palm kernel oils: a randomized blinded crossover study

**DOI:** 10.1186/1471-2431-13-215

**Published:** 2013-12-24

**Authors:** Maria Efigênia de Queiroz Leite, John Lasekan, Geraldine Baggs, Tereza Ribeiro, Jose Menezes-Filho, Mariana Pontes, Janice Druzian, Danile Leal Barreto, Carolina Oliveira de Souza, Ângela Mattos, Hugo Costa-Ribeiro

**Affiliations:** 1 Fima Lifshitz Research Center at Complexo Hospitalar Universitário Professor Edgar Santos, Universidade Federal da Bahia, Salvador, Bahia, Brazil; 2 Pediatric Nutrition R&D, Abbott Nutrition, Abbott Laboratories, Columbus, Ohio, USA; 3 Federal University of Bahia, School of Pharmacy, Salvador, Bahia, Brazil

**Keywords:** Palm olein, Calcium balance, Fat balance, Gastrointestinal tolerance, Brazilian infants

## Abstract

**Background:**

Effects of palm olein (POL) on calcium and fat metabolic balance and gastrointestinal (GI) tolerance have been clinically evaluated but its use in combination with palm kernel oil (PKO), and canola oil has not been similarly assessed in infants.

**Methods:**

Calcium and fat balance and GI tolerance were evaluated in 33 healthy term infants (age = 68-159d) in a randomized, double-blinded, 14d crossover trial at a day care center in Salvador, Brazil; followed by a 4d hospital ward metabolic balance study in 17 of the male subjects. The study compared two commercially available milk-based powdered formulas in Brazil; one containing POL (44% of total fat), PKO (21.7%) and canola oil (18.5%) as predominant fats (PALM), and the other containing none (NoPALM). Occasional human milk (HM) supplementation was allowed at home.

**Results:**

Formula and HM intakes, and growth were not different (p > 0.05). Calcium absorption (%) for infants fed NoPALM (58.8 ± 16.7%; means ± SD) was higher (p = 0.023) than those fed PALM (42.1 ± 19.2%), but was not significant (p = 0.104) when calcium intake was used as a covariate. Calcium intake was higher (p < 0.001) in NoPALM versus PALM fed infants. However, calcium retention (%) was higher in infants fed NoPALM compared to PALM with (p = 0.024) or without (p = 0.015) calcium intake as a covariate. Fat absorption (%) for NoPALM was greater than PALM fed infants (NoPALM = 96.9 ± 1.2 > PALM = 95.1 ± 1.5; p = 0.020 in Study Period I). Mean rank stool consistency was softer in infants fed NoPALM versus PALM (p < 0.001; metabolic period). Adverse events, spit-up/vomit, fussiness and gassiness were not different (p > 0.05). Formula acceptability was high and comparable for both formula feedings, regardless of HM supplementation.

**Conclusions:**

Term infants fed PALM based formula (containing palm olein, palm kernel and canola oils) demonstrated lower calcium retention and fat absorption, and less softer stool consistency versus infants fed NoPALM based formula. Study suggested formula fat differences may affect GI function in infants.

**Clinical trial registration:**

Clinical Trial.Gov # ( http://www.clinicaltrials.gov): NCT00941564.

## Background

Palm olein (POL) is included in the fat blend of most infant formulas globally to mimic the relative amount of palmitic acid (PA) in human milk [1]. However, the positional distribution of individual fatty acids on the triacylglyceride molecules (which affects fat absorption) differs between vegetable oils and human milk (HM) fat [1-4]. Fatty acids on the sn2-position are absorbed as the soluble 2-monoacylglycerides, and fatty acids on the sn1- and sn3-positions are absorbed as free fatty acids. In vegetable oils, including those used in infant formulas, the long chain saturated fatty acids are located primarily on the sn1- and sn3-positions [5]. After digestion, the free PA and stearic acid (SA) conjugate with calcium to form insoluble calcium soaps [6] resulting in reduced fat and calcium absorption. In human milk, PA and SA are found primarily on the sn2-positions (or beta-positions) on triacylglycerides, and are well absorbed after digestion as 2-monoacylglycerides.

Currently available clinical studies (all done in the US and Europe) have demonstrated that fat and calcium are significantly less well absorbed from infant formulas containing POL as the predominant fat source (40 – 45% of total fat) compared to similar formulas containing no POL [7-12]. Some of these studies [8,9] also reported hard stools in the infants fed the POL-based formulas due to increase in stool calcium soap formation. Hard stools have also been reported in breast-fed infants weaned to a POL-based formula [13]. These studies evaluated POL in combination with other fats such as soy, coconut, high oleic safflower or sunflower oils, but not with PKO, palm oil, or canola oil. Moreover, none of these studies [7-12] evaluated powdered formulations which are the predominant form of infant formulas used globally. It is well known that GI responses in infants can sometimes differ between liquid and powdered formulas because of differences in ingredients and manufacturing process [14]. Furthermore, none of the formulations assessed in these studies [7-12] contained supplemental Docosahexaenoic acid (DHA) and Arachidonic Acid (ARA). Most standard infant formulas in the US and many countries currently have DHA and ARA.

In view of the above, the goal of this crossover study was to assess the comparative calcium and fat metabolic balance and gastrointestinal (GI) tolerance (including stool consistency) in healthy normal term infants fed two commercially available milk-based powdered formulas in Brazil; one containing POL, PKO and canola oils as the major fats versus the other formula, which contains none. Both formulas contain DHA and ARA.

## Methods

### Study design, subjects and ethics

**Figure 1:**
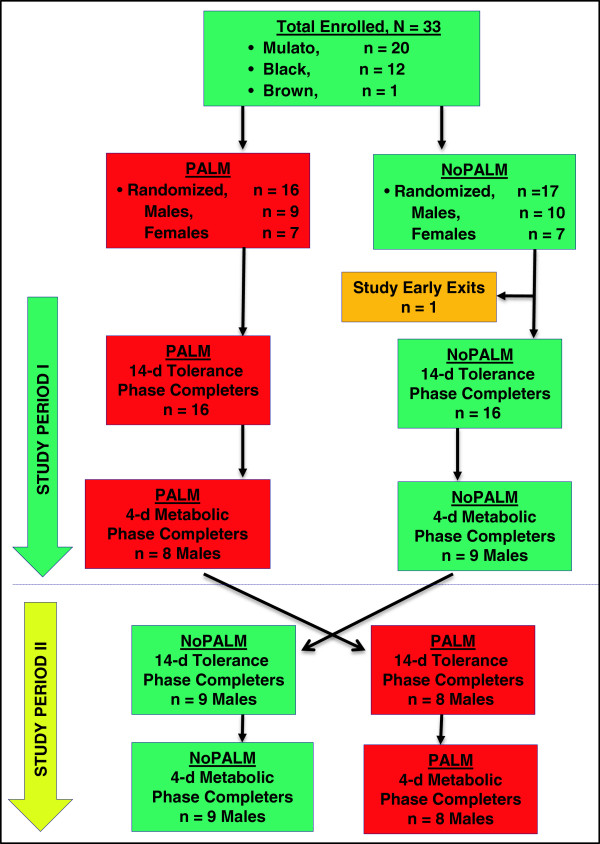
Study flow chart and study subjects’ disposition.

This was a controlled, randomized, two treatments, double-blinded, crossover balance and tolerance study. The trial was conducted in two study periods, periods I and II. Each period had a 14 day tolerance phase and a 4 day metabolic balance phase (Figure 1). The study was approved by the Institutional Research Board at the Federal University of Bahia, Salvador, Brazil and was conducted in accordance with ethical principles that have their origin in the Declaration of Helsinki. The study was also registered with the clinicaltrial.gov (#NCT00941564).

Infant subjects were enrolled and randomized into the study in a day care center in Salvador, Bahia, Brazil. Subjects were full term, healthy male and female infants between 84 and 156 ± 3 days of age at enrollment. They were enrolled into the tolerance phase of the study upon written informed consent from their parents.

### Study feedings

The two study formulas evaluated in this study were commercially available powdered milk-based infant formula in Brazil. One formula contained palm olein (POL), palm kernel (PKO) and canola oils as the predominant fats (PALM; Nestle NAN PRO 1™). The other formula contained no POL, PKO or canola, but had high oleic sunflower (HOS), coconut and soy oils as major fats (NoPALM; Similac Advance™). The NoPALM formula contained higher levels of calcium and phosphorus (Table 1). The two study formulas contained DHA and ARA and comparable levels of vitamin D; and met the levels of nutrients recommended by the Brazil Ministry of Health (ANVISA), Codex Alimentarius [15] and Life Sciences Research Office Expert Panel [16]. Study investigators, subjects and their parents were blinded to the identity of the study formulas. Dedicated study nutritionists handled study formula preparations and coding. The nutritionists were not involved with the care and feeding of infants, data collection and data handling.

**Table 1:** Approximate composition of study formula products (per 100 g of powder)

Nutrient*	**PALM**	**NoPALM**	**Brazil human milk**
			**Reference**^**a**^
Energy, kcal	519	513	
Protein, g	9.5	11	
Carbohydrate, g	57.9	55	
Fat, g	27.7	28	
Palm olein oil (%)	44		
High oleic sunflower oil (%)		41.4	
Palm kernel oil (%)	21.7		
Coconut oil (%)		29.6	
Soy oil (%)		27.6	
Canola oil	18.5		
Corn oil	10.9		
Milk fat	2.8		
Others	2.1^b^	1.4^c^	
Fatty acids (g/100 g Fat)^d^			
16:0	21.95	7.37	17.3 ± 2.2
16:1n-7	0.21	0.09	1.99 ± 0.74
18:0	3.34	3.06	5.3 ± 1.26
18:1n9	40.22	43.22	25.0 ± 3.46
18:2n-6	16.41	19.0	20.3 ± 6.48
18:3n-3	2.02	1.57	1.43 ± 0.66
20:4n-6	0.23	0.42	0.53 ± 0.14
20:5n-3	0.05	0.00	Trace
22:6n-3	0.23	0.16	0.14 ± 0.05
Minerals			
Calcium, mg	279^e^	424^e^	
Phosphorus, mg	160	216	
Magnesium, mg	36	31.0	
Vitamins			
D, μg	7.8	8.6	

*Values are manufacturer’s label claims, except where stated.

^a^Siloa MHL et al. [17].

^b^Docosahexaenoic acid (DHA), Arachidonic acid (ARA) and soy lecithin.

^c^DHA, and ARA.

^d^Analytical values for fatty acids.

^e^Investigator’s analytical values for calcium.

### Evaluation procedures

Infants were enrolled into the study and randomized into one of two feeding groups. The subjects were fed the assigned study formulas exclusively at the day care center during the day on weekdays in the 14-day tolerance phases of periods I and II. However, parents were responsible for feeding the study formulas at home during weekday evenings and nights, and weekends and public holidays. Pre-measured study formula powder and clean potable water were provided to parents with training and clear instructions on formula reconstitution, storage and feeding. Parents agreed to feed the assigned study formula as the primary source of nutrition. However, occasional HM supplementation at home was allowed when parents agreed to weigh the baby and record baby weights before and after breast milk feedings. Parents undertaking the HM supplementation were supplied with weight scales, and were trained to use them.

Daily records of formula intake (volume and frequency), incidence of spit-up and vomiting associated with feedings, occurrence of fussiness, occurrence of gas, and infant’s stool characteristics (frequency, consistency and color) were kept by study personnel at the day care center and by parents at home. Weights of subjects were measured daily at the daycare center and at the hospital by study staffs; however, length and head circumference (HC) were only measured at enrollment/study day 1 and at study day 14 using published standard methods [18,19].

Male subjects who completed the 14-day tolerance phase went through the additional 4-day metabolic phase assessment in both study periods. Female subjects did not undergo the metabolic assessment so as to avoid contamination of stools with urine samples during collection. The male subjects were admitted into the Metabolic Ward at the Fima Lifshitz Metabolic Unit, The Hospital Professor Edgar Santos, Federal University of Bahia, Salvador, Bahia, Brazil, for 4 days. They continued to feed the assigned study formula exclusively throughout the metabolic assessment phase. No breast milk supplementation was allowed during the metabolic assessment. However, mothers of subjects who elected HM supplementation during study tolerance phase continued breast-milk pumping in order to maintain HM stimulation needed to resume HM feeding after study completion. Metabolic assessment was conducted with a 72 hours metabolic sample collection and brilliant blue marker technic using published methods [12,20,21]. Infants were kept on metabolic beds for 3 nights and 4 days. The beds were specially designed to accurately collect separate urine and stool samples throughout the metabolic assessment [19]. Intake, stool and GI tolerance records were collected during the metabolic period. Collected stool and urine samples were stored at -80°C in the laboratory until analyzed. Stool samples were analyzed for fat using the Folch method [22], and study formula samples were analyzed by the method of Bligh and Dyer [23]. Stool and study formula samples were also separately analyzed for calcium using Varian Model 55B atomic absorption spectrophotometer (Varian Medical Systems, Inc., Palo Alto, CA), after acid digestion. Urine samples were analyzed directly using the atomic absorption spectrophotometer without acid treatment. All analyses were done in duplicate.

### Data and statistical analyses

The primary study variable was calcium absorption calculated from calcium intake, and fecal and urinary calcium. The secondary variables included calcium retention, fat absorption, mean rank stool consistency (MRSC scored as 5 = watery, 4 = loose/mushy, 3 = soft, 2 = formed, 1 = hard), and average number of stools per day. The supportive variables included daily study product intake (average volume and average number of feedings), daily human milk intake, percent of feedings with spit up/vomit associated with (within one hour) feeding per day; predominant stool consistency and color, percentages of stool consistency and color, occurrence of fussiness, occurrence of gas, and weight, length and HC; parental responses to the formula satisfaction questionnaire; and study drop-out rate. Safety data included adverse events (AEs) and serious adverse events (SAEs).

Mixed models for carryover data were used to analyze metabolic balance outcomes. Tests for carryover effects were two-sided, 0.10 level tests while tests for feeding and period effects were two-sided, 0.05 level tests. When carryover effects were significant, only Period I data results are valid. During the tolerance phase of the trial, continuous data were analyzed using analysis of variance and categorical data were analyzed using chi-square or Fisher’s exact test. The arcsine of the square root transformation was used for variables expressed as a percentage. Statistical analysis was performed using the SAS® software version 9.1.3 (SAS Institute, Cary, NC). A sample size of 12 subjects (6 per sequence A to B and B to A) has 80% power to detect a difference of at least 15% in calcium absorption, assuming a standard deviation of 7.9%. Approximately 8 subjects per sequence were enrolled to account for 25% attrition. In the tolerance phase, a sample size of 20 subjects per group has 80% power to detect a difference of at least 0.55 in mean rank stool consistency.

## Results

### Disposition, demographic, baseline characteristics and anthropometry of subjects

A total of 33 subjects were enrolled and randomized (PALM = 16; NoPALM = 17) into the study and contributed to the tolerance phase data (Figure 1). One subject on NoPALM feeding had a SAE hospitalization with pneumonia and exited the study prematurely. Thirty two subjects completed the tolerance phase with 23 subjects (PALM = 11; NoPALM = 12) consuming the assigned study formula as the predominant source of nutrition. Of these 23 subjects, 17 male subjects fed assigned study formulas exclusively (no HM feeding) at the Hospital Ward for 4 days. They provided both tolerance and metabolic data during the crossover metabolic phase of the study (PALM =17; NoPALM = 17).

There were no significant differences (p > 0.05) between the two feeding groups in study entry information, study completion rate, adverse events (AEs & SAEs), and other demographic data (Table 2). The age of study subjects ranged from 68 to 159 days and was not significantly different (p > 0.05). Male subjects were 56.3% of the PALM fed group compared with 58.8% of the NoPALM fed group but gender was not different. Birth weight, length, head circumference and gestational age were not different between the two feeding groups (p > 0.05).

**Table 2:** Study entrance information for subjects*

**Variables**	**PALM**	**NoPALM**	**p-values**
Gestational age (weeks)	39.8 ± 1.2 (16)	39.5 ± 1.1 (17)	0.481
Mode of birth, vaginal/cesarean, (n/%)	12/4 (75/25)	11/6 (65/35)	0.708
Birth weight (g)	3333 ± 490 (16)	3321 ± 330 (17)	0.932
Birth length (cm)	49.1 ± 3.7 (15)	49.1 ± 1.9 (17)	0.965
Birth head circumference (cm)	33.7 ± 1.8 (15)	34.2 ±1.3 (15)	0.425
Age at study entrance (days)	117 ± 26 (16)	108 + 27 (17)	0.346

*Values are means ± SD (n).

There were no differences (P >0.05) in weight, length and HC and their interval gains between the two formula feedings during the study. Weights for male subjects in the PALM and NoPALM fed groups at study day 1 were 7008 ± 777 g (means ± SD) and 6934 ± 951 g; and those for female subjects were 6791 ± 1480 g and 6550 ± 1623 g, respectively. The 14 day weight gains for Male subjects in the PALM and NoPALM fed groups were 295 ± 33 g and 374 ± 51 g; and for female subjects were 203 ± 46 g and 282 ± 70 g, respectively.

### Metabolic balance assessment

#### Calcium intake, absorption and retention

**Figure 2:**
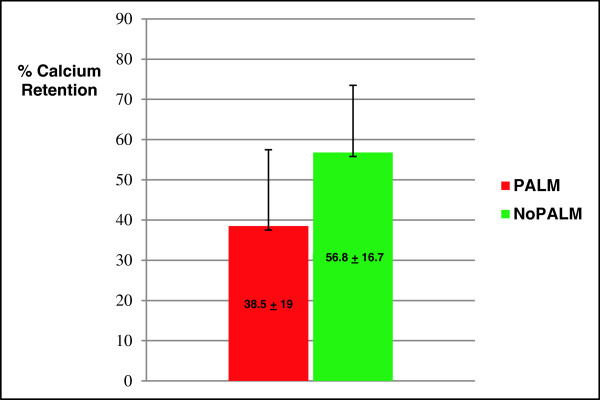
**Calcium retention (%).** NoPALM feeding had a significantly higher calcium retention (% Means ± SD) versus PALM feeding with (p = 0.024; n = 17) or without (p = 0.015) calcium intake as covariate.

There were no significant carryover effects in the calcium metabolic data (Table 3). The intake of calcium was significantly greater with the NoPALM feeding compared to the PALM feeding (p < 0.001); however, there were no differences in stool calcium and urinary calcium. The percent calcium absorption, which was the primary study variable was significantly higher in the NoPALM versus PALM feeding group (p = 0.023), but the significance disappeared (p = 0.104) when calcium intake was used as a covariate in the analyses. However, percent calcium retention was higher in the NoPALM versus PALM feeding groups (p = 0.015), and remained higher (p = 0.024) even when calcium intake was used as a covariate in the analyses (Figure 2).

**Table 3:** Intake and absorption of calcium and fat*

**Variables**	**Metabolic crossover phase**	
	**PALM**	**NoPALM**
	**(n = 17)**	**(n = 17)**
CALCIUM		
Calcium Intake, mg/kg/day	48.3 ± 8.9	72.7 ± 11.8^a^
Stool calcium, mg/kg/day	27.9 ±10.1	30.3 ± 14.7
Calcium absorbed, mg/kg/day	20.4 ± 9.6	42.4 ± 14.6^b^
Calcium absorption, %	42.1 ±19.2	58.8 ± 16.7^c^
Urinary calcium, mg/kg/day	1.69 ± 0.85	1.43 ± 0.77
Calcium retention, mg/kg/day	18.7 ± 9.4	41.0 ± 14.5^a^
FAT		
Fat intake, g/kg/day	4.7 ± 0.9	4.5 ± 0.7
Stool fat, g/kg/day	0.22 ± 0.08	0.14 ± 0.06^d^
Fat absorbed, g/kg/day	4.52 ± 0.85	4.33 ± 0.73
Fat absorption, %	95.1 ± 1.5	96.9 ± 1.2^e^

*Values are means ± SD.

^a^ = p <0.001; ^b^ = p = 0.009; ^c^ = p = 0.023, but using calcium intake as covariate yielded p = 0.104; ^d^ = Significant carryover effect (p = 0.071), therefore, valid period I significant difference (p = 0.027).

^e^ = Significant carryover effect (p = 0.059), therefore, valid period I significant difference (p = 0.020).

#### Fat intake and absorption

There were no significant differences in fat intake between the formula feeding groups (p > 0.05) (Tables 3). There was a significant carryover effect (p = 0.071) noted in the stool fat content; consequently, only study period I results were regarded as valid. At study period I, the stool fat content was significantly lower with the NoPALM feeding compared with the PALM feeding (p = 0.027). Similarly, there was a significant carryover effect (p = 0.059) noted with the percent fat absorption; thus, the results of the study period I were the only valid results. The NoPALM feeding group had a statistically (p = 0.020) higher % fat absorption (~97%) versus the PALM feeding group (~95%).

### Formula gastrointestinal tolerance and acceptability

There were no significant differences (p > 0.05) in study formula intake or HM intake between the two feeding groups (Table 4). There was no HM intake during the study metabolic phase; whereas, the majority of subjects had HM supplementations during the study metabolic phase. The average numbers of feeding per day were significantly higher with the NoPALM feeding compared with the PALM feeding during the tolerance phase, but were not different during the metabolic phase. The occurrences of spit-up/vomit, fussiness and gassiness were not different between the two study formula feedings throughout the study. MRSC, the major GI study variable was softer with the NoPALM feeding compared with the PALM feeding (p <0.001) during the metabolic phase; however, stools were less frequent with the NoPALM feeding (p < 0.036). The percentage of loose/mushy stools was significantly higher (p = 0.002) during the tolerance phase and the percentage of formed stools was significantly lower (p < 0.001) during the metabolic phase with the NoPALM feeding compared with the PALM feeding group, respectively. MRSC was positively correlated with percent loose/mushy stools (r = 0.80; p < 0.001), percent watery stools (r = 0.39; p = 0.024) and percent stool moisture content (r = 0.35; p = 0.043). The predominant color of the stools was yellow and was not different between the two formula feedings. Formula satisfaction questionnaire responses by parents of subjects in the study revealed that both study formulas were similarly well tolerated.

**Table 4:** Formula intake, human milk intake, gastrointestinal tolerance, and stool characteristics*

	**Metabolic period**		**Tolerance period**	
**Variables**	**PALM**	**NoPALM**	**PALM**	**NoPALM**
	(n = 17)**	**(n = 17)**	**(n = 16)**	**(n = 17)**
Average study formula intake, mL/kg/d	113 ± 20	114 ± 19	99 ± 31	103 ± 33
Average human milk intake, g/kg/d	0 ± 0	0 ± 0	94 ± 36 (n = 15)	81 ± 29 (n = 12)
Average numbers of feedings, #/d	6.3 ± 0.2	6.2 ± 0.1	4.6 ± 0.6	5.4 ± 0.9^d^
Spit-up/vomit, % of feedings	4.0 ± 4.8	3.5 ± 4.7	12.8 ± 25.5	13.9 ± 21.4
Stool frequency, # stools/d	3.4 ± 0.7	2.9 ± 0.9^a^	1.7 ± 0.6	1.9 ± 0.7
Mean rank stool consistency score (MRSC)^‡^	2.4 ± 0.3	3.0 ± 0.5^b^	3.3 ± 0.5	3.5 ± 0.6
Stool consistency, % of stools				
Watery	0 ± 0	0.5 ± 2	27.5 ± 19	26.9 ± 19.9
Loose/mushy	1.2 ± 3.5	19.2 ± 21.6^c^	19.1 ± 17.2	38.2 ± 14.2^e^
Soft	39.7 ± 26.5	58.9 ± 30.8	43.7 ± 18.9	32.6 ± 23.8
Formed	59.1 ± 25.7	21.4 ± 35.8^a^	21.6 ± 12.5	20.7 ± 16.3
Hard	0 ± 0	0 ± 0	10.8 ± 4.9	0 ± 0
Total stool production, g/kg/day	5.2 ± 2.3	4.2 ± 2.7		
Stool moisture content, %	82.8 ± 2.6	84.8 ± 6.5		

*Values are means ± SD.

**Sample sizes (n) for each column are in parenthesis except where noted to be different.

^‡^MRSC score: 5 = watery, 4 = loose/mushy, 3 = soft, 2 = formed, 1 = hard (higher is softer).

^a^ = p <0.036; ^b^ = p = 0.001; ^c^ = Significant carryover effect (p = 0.077) Period I only, PALM = 0.9 ± 2.8 & NoPALM = 8.3 ± 11.7 (NS at p = 0.122); ^d^ = p = 0.005; ^e^ = p = 0.002.

## Discussion

The results of the current study successfully demonstrated that calcium metabolic balance (absorption or retention) was significantly higher with the NoPALM formula feeding compared to the PALM formula feeding (containing POL, PKO and canola oils). The results are consistent with those obtained in previous studies [7,11] comparing calcium absorption and/or retention from POL predominant milk-based formulas versus non-POL containing formulas. The POL formulas evaluated in those previous studies contained other vegetables oils instead of PKO or canola oils. Consequently, the effects of POL predominant formulas on calcium absorption are consistent, irrespective other combinations of oil with POL. The calcium absorption value of 58.8% versus 42.1% for the NoPALM (non-POL) versus PALM (POL) formula noted in the current study is consistent with the values of 57.4% versus 37.5% for the non-POL versus POL formula reported by Nelson et al. [7]. Similarly, the relative calcium retention value of 56.8% versus 38.5% for the NoPALM versus PALM formula shown in our current study is directionally consistent with the value of 47.2% versus 32.5% for the non-POL versus POL formula previously reported in subsequent study by Nelson et al. [11]. The fat balance results are also comparable to those reported in the previous studies [7,11]. Notwithstanding the differences in fat blend compositions (with or without PKO, canola, DHA and ARA oils) and formula types (powder versus liquid) of the formulas evaluated in our current study versus previous studies, the findings regarding the impact of POL predominant formulas on calcium and fat balance are similar.

Historically, the percent calcium bioavailability from infant formulas was assumed to be about 38% versus 58% for human milk [24,25]. Consequently, higher levels of calcium were added to infant formulas, especially soy-based formulas and specialized formulas so as to compensate for their lower calcium bioavailability. However, not all infant formulas typically have a lower calcium bioavailability. Standard milk-based formulas containing no POL have been shown [7,11] to have a calcium bioavailability that is closer to that for human milk (HM). The calcium bioavailability from the PALM formula in the current study is close to the average value of 58% reported for HM [24]. Furthermore, the percent fat absorption noted in the present study was greater in the NoPALM group compared to the PALM group. The percent fat absorption (~95 – 97%) from both study formula groups was good and comparable to that reported from HM [26].

The relevance of the higher calcium balance noted with the NoPALM versus PALM feeding in the current study is not diminished by the observed impact of calcium intake on the percent calcium absorption when used as a covariate. First, calcium absorption has been suggested to be enhanced at lower calcium concentration and reduced at higher concentration [27,28]. Nonetheless, calcium absorption will also depend on many other factors as well. It is well accepted that calcium absorption from HM is significantly higher than that from many infant formulas despite the lower level of calcium in HM and the consequential lower intake of calcium from HM [24,25]. Obviously, the lower level of calcium in the PALM in this study did not result in enhanced calcium absorption as in HM. Secondly, the significantly lower percent calcium retention noted with the PALM feeding was maintained after adjusting for calcium intake as a covariate in the analyses of variance. This suggests that the differences in the fat blends of the study formulas have relevant impact on the calcium balance noted in the study. Calcium retention and bone mineral content are better markers or functional outcomes for the impact of dietary calcium on calcium homeostasis compared to calcium absorption [29]. A recently published study [30] demonstrated a significantly (p = 0.041) lower bone mineral content at 3 months of age in term infants fed a POL containing partially hydrolyzed whey protein-based formula compared to a similar formula containing no POL. The fat blends of formulas compared in that study were similar to the PALM and NoPALM formulas assessed in our current study except for the inclusion of PKO and canola in the PALM formula of our current study.

There were notable differences in the GI tolerance of the study formulas by the subjects in the current study. MRSC was soft for the NoPALM feeding group, and formed for the PALM feeding group (Table 4). The stool consistency results in this study were similar to those observed in studies assessing the GI tolerance to formulas containing POL and those without POL. Studies have reported an increase in the formation of calcium soap-containing hard/firm stools in infants fed formula with POL as the predominant oil compared to infants fed either similar formulas containing no POL or infants fed HM [8,9,12,13].

The original intent of the current study was to allow minimal HM supplementation when the infant subjects were at home and not at the day care center or at the Hospital Metabolic Ward during this study. This was to accommodate nights, weekends and holidays. Despite the provision of study formulas to parents for home feeding, the feeding of human milk was substantial in this study. However, the HM supplementation intake was not different between the two formula feeding groups (Table 4). Previous metabolic studies on POL predominant formulas (7–12) which were all done in the US or Europe did not report extensive HM supplementation. Nonetheless, it was interesting and reassuring that differences in clinical outcomes were reasonably obtained from feeding different types of formulas, even when HM supplementation was substantial. The results of the current study suggested that infant formula fat blend differences may affect calcium and fat balance and GI stool tolerance in formula fed infants receiving HM supplementations. However, this study did not address the amount of formula feeding or HM supplementation that might possibly eclipse the differences noted in the current study.

## Conclusions

In this study, the NoPALM powdered formula, which was free of POL or PKO or canola, demonstrated significantly higher calcium retention, and a higher fat absorption in healthy term infants, compared to the PALM powdered formula, which contained palm olein (POL) palm kernel (PKO) and canola oils as the predominant fat, regardless of HM supplementation. The NoPALM fed infants also demonstrated a softer stool consistency compared to PALM fed infants; however, both PALM and NoPALM fed infants generally demonstrated comparable normal GI tolerance and acceptability despite the HM supplementation. The results of this study in Brazilian infants are consistent with the results of previous studies on POL predominant formula despite differences in other fats combined with POL, the type of formula (powder versus liquid) and HM supplementation. In conclusion, results of this study suggest that differences in fat blends used in infant formulas may affect calcium and fat metabolic balance, and GI stool tolerance in term infants.

## Abbreviations

AN: Abbott Nutrition, Abbott Laboratories; AE: Adverse event; ARA: Arachidonic acid; DHA: Docosahexaenoic acid; EVP: Exploratory evaluable population; GI: Gastrointestinal; HM: Human milk; HC: Head circumference; MRSC: Mean rank stool consistency; NoPALM: Milk-based powdered commercial formula containing no palm olein; PA: Palmitic acid; PKO: Palm kernel oil; PALM: Milk-based powdered commercial formula containing palm olein, palm kernel and canola oils as predominant fats; POL: Palm olein; SAE: Serious adverse effect; SA: Stearic acid.

## Competing interests

This study was funded by AN, Abbott Laboratories. Two authors (JL and GB) are employees of AN, Abbott Laboratories. Author (HC) received research funding from AN, and other infant formula companies and speaks at programs sponsored by AN.

## Authors’ contributions

All authors participated in the development, review and approval of the manuscript. Author HC is the principal study investigator and senior author. Authors HC, TR and JL participated in every aspects of the study. Author GB participated in the design, statistical analyses and development of the manuscript. Authors ML, JM, JD, MP, DB, CS and AM participated in conducting the clinical trial, and collection and analyses of study samples.

## Pre-publication history

The pre-publication history for this paper can be accessed here:

http://www.biomedcentral.com/1471-2431/13/215/prepub
